# Primary pleural angiosarcoma as a mimicker of mesothelioma: a case report **VS**

**DOI:** 10.1186/1746-1596-6-130

**Published:** 2011-12-30

**Authors:** Yu-Chien Kao, Jyh-Ming Chow, Kum-Min Wang, Chia-Lang Fang, Jan-Show Chu, Chi-Long Chen

**Affiliations:** 1 Department of Pathology, Wan Fang Hospital, Taipei Medical University, Taiwan; 2 Division of Hematology and Medical Oncology, Department of Internal Medicine, Wan Fang Hospital, Taipei Medical University, Taiwan; 3 Division of Thoracic and Cardiovascular Surgery, Department of Surgery, Wan Fang Hospital, Taipei Medical University, Taiwan; 4 Department of Pathology, Taipei Medical University Hospital, Taiwan

**Keywords:** angiosarcoma, pleura, mesothelioma, sarcoma, pathology

## Abstract

Primary pleural angiosarcoma is a rare and clinically aggressive tumor. Patients usually present with chest pain, dyspnea, hemoptysis and/or cough. Radiologic studies reveal diffuse pleural thickening and pleural effusion with or without mass lesion. The clinical and radiological features both resemble those of mesothelioma, and its definite diagnosis requires careful histologic examination. However, frequent epithelioid feature and immunoreactivity to cytokeratin in primary pleural angiosarcoma further complicate the pathologic diagnosis. The use of proper immunohistochemical stains is often needed to support endothelial differentiation in the tumor cells and to exclude metastatic carcinoma and mesothelioma. We report the case of a 49-year-old male patient with primary pleural angiosarcoma, who presented with initial hemothorax, followed by a rapid progress to an inoperable status.

## Background

Angiosarcoma is an uncommon malignant tumor of endothelial differentiation. It accounts for about 1% of all soft tissue malignancies and most commonly arises in skin, soft tissue, breast, liver, bone and spleen [1].

Primary pleural angiosarcoma (PPA) is a rare occurrence. Since its first description in 1943,[2] only 39 reported cases of PPA have been published [1-22]. Patients almost always die of the disease within months. Definite diagnosis is usually not possible using the examination of cytology or small biopsy specimens, but often requires that of decortication or resection specimens. The histologic picture of biphasic spindle and epithelioid tumor cells along with immunoreactivity to epithelial markers, such as cytokeratin and CK7, may lead to an erroneous diagnosis of mesothelioma or metastatic sarcomatoid carcinoma. Identifying areas showing vasoformative tendency and immunostains with endothelial markers, such as CD31, CD34, factor VIII and FLI-1, are diagnostically important.

We describe herein a case of PPA, to highlight its aggressive clinical behavior and the diagnostic pitfalls.

## Case presentation

### Clinical summary

**Figure 1:**
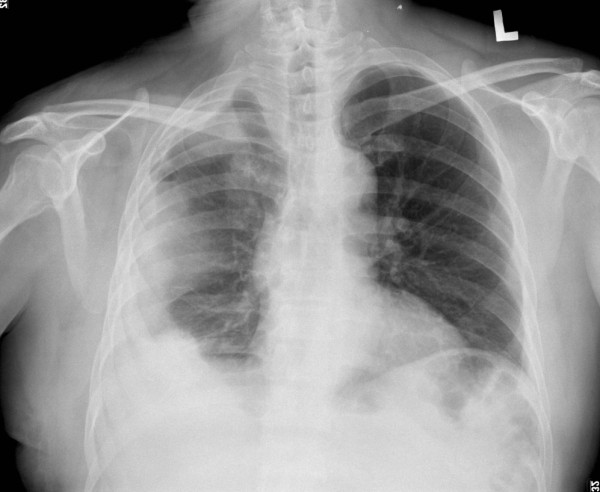
**Chest roentgenogram**. Plain chest film showed right-side loculated pleural effusion.

A 49 year-old-male patient presented with intermittent right chest pain for one month. The pain progressed with exertional dyspnea. He had a 10-year history of asthma under regular medical treatment and was an ex-smoker (half package-per-day for 20 years in the past) who quit 10 years ago. He did not have any history of asbestos exposure or tuberculous infection. In physical examination, breathing sounds were decreased at the right lung. Chest radiography and computed tomography revealed right-side loculated pleural effusion with pleural thickening but without mass lesion (Figure 1). Fine needle aspiration showed some bloody and sticky pleural effusion. The value of hematocrit of the effusion was 39.5% (peripheral blood hematocrit: 38.9%). Cytologic examination of the pleural effusion showed negative for malignancy. Thoracoscopic examination revealed diffuse blood clot coating and thickening of the visceral and parietal pleura over the right hemithorax. He received thoracoscopic decortication of the pleura. Subsequent staging image series showed no evidence of metastatic lesion in brain, bone and lung.

One month later, open thoracotomy revealed tumor mass extending along the previous surgical wound and encasing the right pulmonary hilum. Curative resection could not be done, and the patient received an excisional biopsy of the tumor.

### Pathologic findings

**Figure 2:**
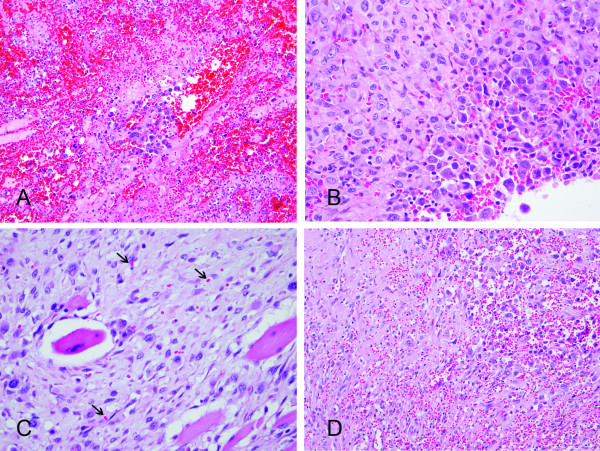
**Histomorphology of the decortication specimen**. Pleural angiosarcoma with scattered tumor cells in the hemorrhagic background (A). Solid sheets of epithelioid tumor cells can simulate mesothelioma and/or carcinoma (B). Intracytoplasmic lumen containing red blood cells (C, arrowhead) and anastomosing vascular spaces (D) are evidence of endothelial differentiation (hematoxylin-eosin, ×200 [A, D] and ×400 [B, C]).

**Figure 3:**
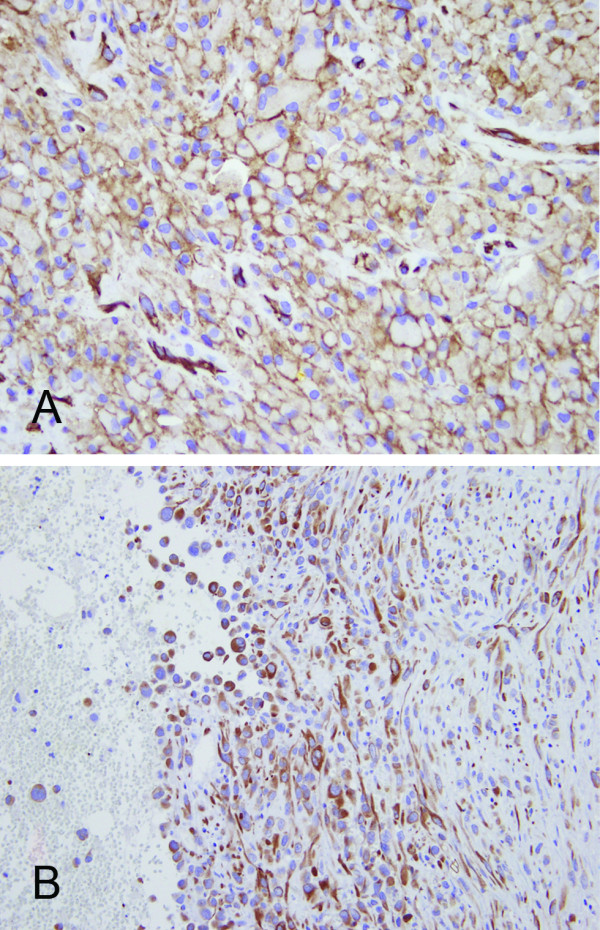
**Immunohistochemical stains**. Diffuse CD31 positivity (A) indicates the vascular nature of the tumor cells. Diffuse immunoreactivity for cytokeratin stain (B) could be a diagnostic pitfall. (×400 [A] and ×200 [B]).

Histologically, the specimens showed an extensive hemorrhagic background with epithelioid tumor cells arranged in solid sheets and discohesive cells separated by abundant red blood cells (RBCs) (Figure 2A-B). Infiltrative growth of the tumor cells in a hyalinized to fibrous background and between skeletal muscles was also present. The tumor cells had pleomorphic nuclei, irregular nuclear contour, occasional binucleation, vesicular chromatin, prominent nucleoli and abundant eosinophilic cytoplasm, giving an epithelioid feature. Rudimentary and anastomosing vascular channels and Intracytoplasmic lumens containing RBCs were seen in areas (Figure 2C-D). Spindle tumor cells were observed as a minor component of the tumor. The results of immunohistochemical stains showed diffuse positive staining for CD31, FLI-1, cytokeratin (AE1/AE3) and CK7 (Figure 3A-B), whereas CD34, factor VIII, and mesothelial markers including calretinin, CK5/6, HBME-1, and WT-1 were negative. An angiosarcoma was diagnosed. The patient then received radiotherapy (3750 cGy with 15 fractions), five cycles of chemotherapy with mesna, ifosfamide, doxorubicin and dacarbazine, two cycles of cisplatin and doxorubicin and three cycles of doxorubicin, etoposide and thalidomide. He was alive with the disease nine months after the diagnosis was made.

## Discussion

PPA is an aggressive and rapidly fatal disease. Only 39 cases have been reported in the literature[1-22] after excluding epithelioid hemangioendothelioma. Together with our patient, the ages of the patients ranged from 33 to 79 years (mean: 59.1 years) with male predominance (4:1, Figure 4). The most common presentation is chest pain (47.5%), followed by dyspnea (35%), hemoptysis (27.5%), cough (15%) and weight loss (10%). Radiologic examinations usually show unilateral or bilateral effusion (70%) and diffuse pleural thickening (40%), simulating the presentation of mesothelioma. Mass lesions are seen in about half of the patients (54.3%) [2-7,9,10,14,20-22].

**Figure 4:**
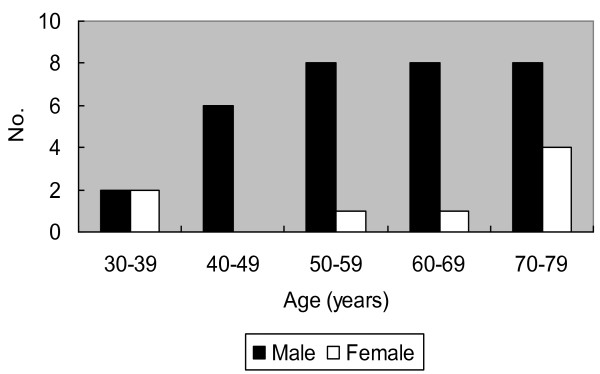
**Age and sex distribution of reported cases of primary pleural angiosarcoma**. Male predominance was shown in the age group of 40-79 years.

The effusions are usually bloody. Cytologic examination of the pleural effusion is rarely helpful in making the diagnosis. In the seven cases with available cytologic examination results,[1,8,9,13,19,20,22] presence of atypical cells was described in only one case [20]. Most patients were diagnosed by surgical excision (32.5%, including debulking excision, pleurectomy and decortication) [3,5,7,9,12,13,15,17,18,20] or autopsy in rapidly lethal cases (27.5%) [2,4,5,8,10,21,22]. Using non-invasive biopsy and surgical biopsy, the patients were diagnosed as PPA in a smaller proportion of cases (17.5% and 10%, respectively) [1,5,6,14,16,19]. Six out of 40 patients received a non-diagnostic biopsy [8,9,13,19,21,22]. Thus, in the context of refractory bloody pleural effusion of uncertain etiology, we suggest that surgical biopsy or excision should be considered even without mass lesion.

The histologic features show malignant spindle and/or epithelioid neoplasm with areas of vasoformative nature, such as vascular spaces lined by atypical tumor cells and intracytoplasmic lumen containing RBCs. Epithelioid features were mentioned in the majority of cases (72.5%) and constituted variable proportion of the tumors [1,5-15,17-19]. The differential diagnosis of a biphasic pleural tumor mainly includes mesothelioma and sarcomatoid carcinoma. In general, mesothelioma shows more monotonous tumor cells with less degree of cytologic atypia. Intracytoplasmic lumen can be seen both in angiosarcoma and mesothelioma, but intraluminal RBCs are not seen in mesotheliomas [11]. Sarcomatoid carcinoma less likely manifests as diffuse pleural involvement. The carcinomatous component and clinical history would aid in diagnosis.

Immunohistochemical stains have an important role in differential diagnosis. Expression of at least one of the endothelial markers including CD31, CD34, factor VIII and FLI-1, is required to confirm the diagnosis of angiosarcoma. Among them, CD31 is considered to be the most sensitive and specific [1]. Epithelial markers can be expressed in angiosarcoma, especially in the epithelioid variant [23]. Positive cytokeratin staining was found in 60.9% cases of PPA (14 of 23, including our patient) [1,5,11,14,15]. Immunoreactivity to CAM5.2, CK7, CK8 or CK18 has also been reported [1,13,14]. Epithelial membrane antigen was negative in all those 14 cases with available results [5,6,12,16,19,20]. The expression of epithelial markers can be misleading in pathologic diagnosis. One should bear in mind that cytokeratin is also frequently expressed in epithelioid vascular tumors. Although the expression pattern of cytokeratin can range from diffuse and strong to focal and weak in distribution, it is usually not as strong as that in carcinoma or mesothelioma [17]. Mesothelial markers such as calretinin, CK5/6, HBME-1 and WT-1, are used to exclude malignant mesothelioma.

The etiologic factors in most cases of PPA are still unknown. Some case reports from Japan indicated the relationship with tuberculous pyothorax (n = 11), [5,15,21] while some cases from western countries had history of asbestos exposure (n = 4)[7,10] or radiotherapy (n = 1) [11]. Association with tuberculosis has been first described by Myoui et al. in 3 out of 4 PPA patients [24]. Based on a series of autopsy cases, Aozasa et al. later reported six additional PPA patients associated with tuberculosis and postulated 3,600-fold increased risk of PPA in pyothorax patients [5]. But, Hattori et al. suggested that pyothorax-associated angiosarcoma often manifest as chest wall soft tissue tumors instead of diffuse pleural involvement and should be separated from PPA [25]. Attanoos et al reported three PPA patients with history of asbestos exposure [10]. However, only one of them had typical asbestos bodies in the lung and increased asbestos fiber content by mineral analysis. One patient with history of previous radiotherapy for ovarian carcinoma developed simultaneous pleural and peritoneal angiosarcoma [11]. Our patient did not have any history of tuberculosis, asbestos exposure or radiotherapy.

**Figure 5:**
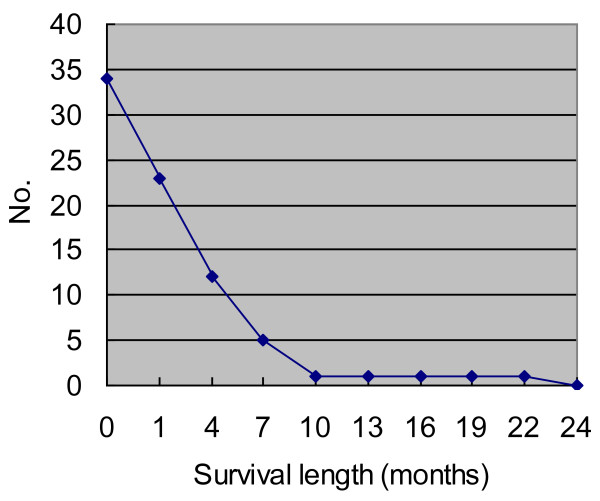
**Survival length of reported available 34 cases of primary pleural angiosarcoma**. Most patients died of disease within several months, and all of them died by the end of 24 months.

The treatments of angiosarcoma include surgical excision, radiotherapy and/or chemotherapy. However, the clinical course is usually rapidly fatal, regardless of treatment modalities. Seventy percent of the patients died of disease within 7 months (Figure 5). The prognosis is even worse than angiosarcoma of other organs [23]. According to the occurring frequency, metastatic sites reported in the literature include lymph node, adrenal gland, bone, brain, oral cavity, liver, skin, spleen, gastrointestinal tract, kidney and spinal cord [2,3,5,8,15,18,19]. In addition to nodal metastasis (n = 6) as in epithelioid angiosarcoma of other organs,[23] autopsied cases of PPA reported by Aozasa et al. show preference of adrenal gland metastasis (n = 5) [5].

## Conclusion

In conclusion, PPA is a highly malignant disease, which may mimic mesothelioma clinically, radiologically and even pathologically. The diagnostic pitfalls include mesotheliomatous growth pattern with diffuse pleural involvement, biphasic histomorphologic pattern and immunoreactivity to cytokeratin. An accurate diagnosis requires careful pathologic examination to identify the evidence of endothelial differentiation aided with immunohistochemical stains.

## Consent

Written informed consent was obtained from the patient for publication of this Case Report and any accompanying images. A copy of the written consent is available for review by the Editor-in-Chief of this journal

## List of abbreviations used

PPA: primary pleural angiosarcoma; RBC: red blood cells.

## Competing interests

The authors declare that they have no competing interests.

## Authors' contributions

YCK participated in drafting the manuscript and literature review. JMC and KMW were responsible for acquisition of clinical data, follow-up information and the surgery (KMW). CLF, JSC and CLC participated in making the histopathological diagnosis, conception of the idea and revising the manuscript. All authors have read and approved the final manuscript.
